# Development of a Web-based Family Intervention for BRCA Carriers and Their Biological Relatives: Acceptability, Feasibility, and Usability Study

**DOI:** 10.2196/cancer.9210

**Published:** 2018-04-13

**Authors:** Maria C Katapodi, Miyeon Jung, Ann M Schafenacker, Kara J Milliron, Kari E Mendelsohn-Victor, Sofia D Merajver, Laurel L Northouse

**Affiliations:** ^1^ Department of Public Health Faculty of Medicine University of Basel Basel Switzerland; ^2^ School of Nursing University of Michigan Ann Arbor, MI United States; ^3^ School of Nursing Indiana University Indianapolis, IN United States; ^4^ Comprehensive Cancer Center University of Michigan Ann Arbor, MI United States; ^5^ Medical School University of Michigan Ann Arbor, MI United States; ^6^ School of Public Health University of Michigan Ann Arbor, MI United States

**Keywords:** BRCA families, family-based intervention study, Web-based intervention study, psycho-educational and skills-building intervention study, communication and coping, patient decision-aid, genetic testing

## Abstract

**Background:**

Carriers of breast cancer gene (*BRCA*) mutations are asked to communicate genetic test results to their biological relatives to increase awareness of cancer risk and promote use of genetic services. This process is highly variable from family to family. Interventions that support communication of genetic test results, coping, and offer decision support in families harboring a pathogenic variant may contribute to effective management of hereditary cancer.

**Objective:**

The aim of this paper was to describe the development of the Family Gene Toolkit, a Web-based intervention targeting *BRCA* carriers and untested blood relatives, designed to enhance coping, family communication, and decision making.

**Methods:**

We present findings from focus groups regarding intervention acceptability and participant satisfaction and from a pre-post pilot study with random allocation to a wait-listed control group regarding intervention feasibility and usability.

**Results:**

The Family Gene Toolkit was developed by a multidisciplinary team as a psycho-educational and skills-building intervention. It includes two live webinar sessions and a follow-up phone call guided by a certified genetic counselor and a master’s prepared oncology nurse. Each live webinar includes two modules (total four modules) presenting information about *BRCA* mutations, a decision aid for genetic testing, and two skill-building modules for effective coping and family communication. Participants in focus groups (n=11) were highly satisfied with the intervention, reporting it to be useful and describing clearly the important issues. From the 12 dyads recruited in the pre-post pilot study (response rate 12/52, 23%), completion rate was 71% (10/14) for intervention and 40% (4/10) for wait-listed control groups.

**Conclusions:**

Acceptability and satisfaction with the Family Gene Toolkit is high. On the basis of the findings from usability and feasibility testing, modifications on timing, delivery mode, and recruitment methods have been implemented.

**Trial Registration:**

ClinicalTrials.gov NCT02154633; https://clinicaltrials.gov/ct2/show/NCT02154633 (Archived by WebCite at http://www.webcitation.org/6yYNvLPjv)

## Introduction

### Background

Women with germline mutations in the *breast cancer (BRCA) type 1* and *BRCA type 2* genes (hereafter *BRCA*) have a 55% to 70% chance of developing breast cancer and 17% to 59% chance of ovarian cancer by the age of 70 years, where the equivalent lifetime risks in the general population are 12% and 1.3%, respectively [1]. These women also have an increased risk for early cancer onset, before screening recommendations apply, and for triple-negative tumors, that is tumors that test negative for estrogen, progesterone, and human epidermal growth factor  *receptor* 2 (HER2) and do not respond to hormonal therapy (eg, tamoxifen) or therapies that target HER2 receptors, (eg, herceptin) [2]. Germline *BRCA* mutations are inherited in an autosomal dominant manner; for every *BRCA* carrier, first, second, and third degree relatives have 50%, 25%, and 12.5% risk, respectively, for inheriting the pathogenic variant [3]. The availability of genetic testing for *BRCA* mutations is a significant milestone for effective cancer control, as blood relatives can be tested with almost 100% accuracy [4]. Genetic counseling and testing provide information about available risk management options (eg, screening at a younger age). Testing also confirms the non-inheritance of an identified mutation, preventing unnecessary early-onset screening in true negative relatives [5].

Underutilization of genetic testing among biological relatives indicates that its potential benefits are not communicated effectively [6-10]. Barriers to family communication include lack of understanding of genetic information, often hampering the ability of the family to cope with health threats associated with the pathogenic variant [11]. Lack of communication skills and lack of effective coping strategies (eg, avoidance) inhibit disclosure of test results to relatives [12,13]. Although helping family members learn more about their cancer risk is a leading motivation among women pursuing genetic testing [14,15], positive test results may also generate conflicts. Poor communication about implications of increased cancer risks associated with the pathogenic variant may leave family members unaware of the need for genetic counseling. Poorly informed decisions motivated by anxiety, fear, exaggerated perceptions of risk, together with lack of knowledge often lead to decisional conflict among biological relatives [16-21]. Interventions supporting disclosure of genetic test results and enhancing helpful coping (eg, information seeking) in mutation-harboring families could contribute to more open communication about cancer risks, informed decisions for genetic testing, and better management of hereditary breast and ovarian cancer (eg, prophylactic mastectomy and salpingo-oophorectomy in mutation carriers).

We identified 32 patient decision aids (PtDAs) targeting women who were confirmed mutation carriers or at risk of carrying a pathogenic variant (Multimedia Appendix 1). These PtDAs have been designed to improve decision making for genetic testing (n=12), decision making for cancer risk management options (n=7), increase understanding of cancer genetics (n=4), enhance active coping and well-being after a pathogenic variant has been identified (n=3), and provide support for disclosing genetic test results to family members (n=6; Multimedia Appendix 1). Commonly examined outcomes were satisfaction with the intervention (n=12), knowledge of breast and ovarian cancer genetics (n=14), intention to use genetic testing and values clarification (n=10), emotional burden (n=12), perceived breast cancer risk and/or risk of carrying a pathogenic variant (n=5), behavioral changes (eg, preventive surgery and exercise; n=6), and family communication for test results (n=4). Outcomes across studies were consistent regarding satisfaction with the PtDA and increased knowledge of breast or ovarian cancer genetics. Findings for other outcomes were often inconsistent.

PtDAs were delivered in several ways, the most common being face-to-face or group-enhanced counseling (n=13), followed by booklet or leaflet or printed material (n=10). Fewer studies used noninteractive CD-ROMs or other computer-based sources (n=5), whereas more recent studies used Web-based, online, interactive modules (n=4). Most PtDAs targeted women after they had been referred for genetic counseling or after confirmation that a *BRCA* mutation had been identified (n=20). Fewer PtDAs targeted biological relatives of mutation carriers or women with strong family history (n=7), and only two PtDAs included both mutation carriers and biological relatives (Multimedia Appendix 1).

*BRCA* mutations affect the whole family, and genetic testing can cause tensions among family members [22,23]. Most of the above PtDAs targeted only mutation carriers and did not include relatives. Communication of genetic results in families is a two-way exchange that takes place between mutation carriers and relatives. It depends on understanding genetic information, communication skills, and coping competencies of everyone involved. Explaining genetic information to biological relatives is most effective when combined with effective coping strategies for cancer risk (eg, seeking expert advice) and decreasing decisional conflict for genetic testing.

To address these gaps, the specific aims of this study were to develop an interactive, Web-based communication, coping, and decision-support PtDA targeting *BRCA* carriers and biological relatives (Family Gene Toolkit); determine the acceptability of the Family Gene Toolkit and participant satisfaction using focus groups; and examine usability and feasibility in a pre-post pilot study. In this paper, we first present the development of the Family Gene Toolkit and then the methods and results of two sequential studies. The first study involved focus groups that assessed acceptability and participant satisfaction. The second study was a pre-post pilot that assessed usability and feasibility.

**Figure 1:**
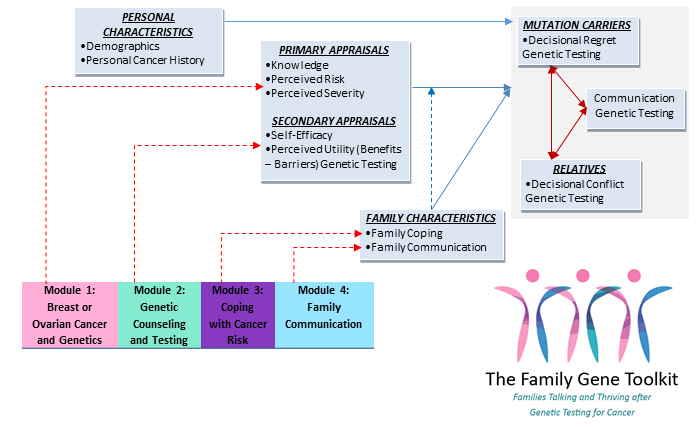
Theoretical framework guiding the development of the Family Gene Toolkit.

### Development of the Family Gene Toolkit

The development of the Family Gene Toolkit and selection of outcomes were based on the theory of stress and coping [24] adapted to reflect the needs of *BRCA* families. The model integrates bio-psychological family adaptation in genetic illness [25], consequences of genetic testing from a stress and coping perspective [26], and decision making and decision support for genetic testing associated with hereditary breast and ovarian cancer [27]. Stress occurs when primary appraisals of a health problem threaten a person’s psychological and physical well-being. Secondary appraisals regarding risks and benefits associated with the health problem and the availability of coping resources can either exacerbate stress or mitigate it. Perceived lack of family support regarding genetic testing may increase stress after a pathogenic variant has been identified, whereas self-efficacy in managing cancer risks may reduce stress. The theoretical framework guiding the study was tested with 168 families at risk for hereditary breast or ovarian cancer [11] (Figure 1).

The Family Gene Toolkit is a psycho-educational and skills-building intervention targeting *BRCA* families. It was developed by a multidisciplinary team, including three expert nurses in psychosocial oncology, communication, and executive cognitive function; a genetic counselor; and a physician expert in *BRCA* mutations. The content was based on empirical findings from a descriptive study with 168 at-risk families [11,28], a meta-analysis of interventions targeting cancer patients and their family caregivers [29], feedback from a psychologist with expertise in decision making for genetic testing who was not involved in the development in the intervention, and feedback from two *BRCA* families (two female carriers and two female relatives). The intervention prototype targets family dyads consisting of a female mutation carrier and a female biological relative.

The Family Gene Toolkit has been designed to address challenges related to the quantity and complexity of genetic information patients are asked to understand and communicate [30,31]. First, understanding the context of hereditary breast and ovarian cancer (HBOC; eg, of mutation, prognosis, prevention, and treatment) is important for decision making. Second, patients’ understanding of the accuracy of the genetic test and the difference between specificity (accurate detection of a variant) and sensitivity (accurately determining that a variant is not present) influences their understanding of how test results will or will not affect decision making about prevention and treatment. Third, genetic diseases are chronic and require ongoing coping and self- management. Patients’ ability to self-manage and actively cope with health challenges should be addressed. Finally, patients’ values and communication skills are important because of family implications.

**Figure 2:**
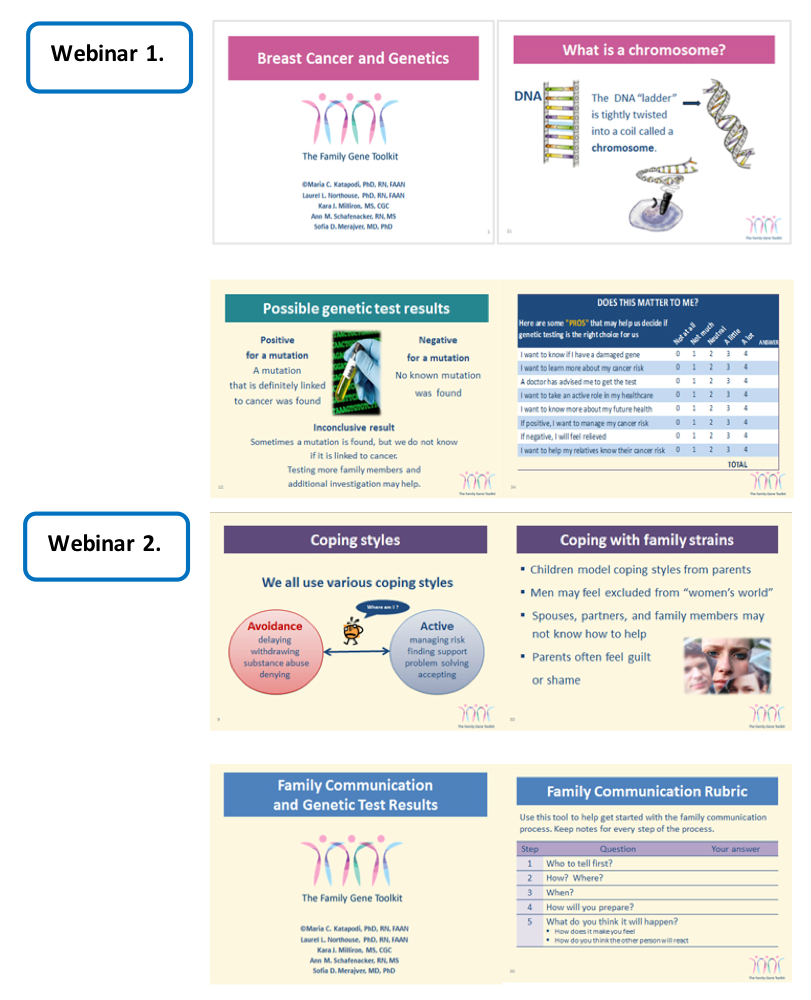
Examples from the four modules of the Family Gene Toolkit.

Considerations of subsequent family communication about genetic cancer risk and personal values are critical. The four modules of the Family Gene Toolkit embrace the above challenges and cover these topics (Figure 2):

- *Module 1: breast cancer and genetics* provide background information about breast cancer development and the role of heredity (module 1A). It explains the epidemiology and probabilities of the disease with and without a germline *BRCA* mutation. A module for *ovarian cancer and genetics* (module 1B) was developed for ovarian cancer patients. Risks associated with other cancers connected to *BRCA* mutations in both genders, ie, prostate and pancreatic cancers and melanoma, are also presented in module 1.
- *Module 2: genetic counseling and testing* provides decisional support for genetic testing to relatives, including a description of the counseling process, potential risks, benefits, limitations of genetic testing, and possible results. It incorporates formal elements of PtDAs based on the International Patient Decision Aids Standards criteria [32] and patient testimonials about accepting or refusing testing.
- *Module 3: coping with cancer risk* discusses common challenges faced by *BRCA* families, including an overview of different coping styles, the importance of active coping, and practical tips to facilitate active coping with different personal and family challenges. It is designed to enhance active coping and family support concerning hereditary cancer risk and includes narratives from mutation carriers to support these points.
- *Module 4: family communication* presents testimonials about the responsibility to share test results, the importance of open family communication about the mutation, common issues that arise during this process, and practical ways to avoid conflicts. It provides a five-steps training designed to enhance communication skills in family members.

**Figure 3:**
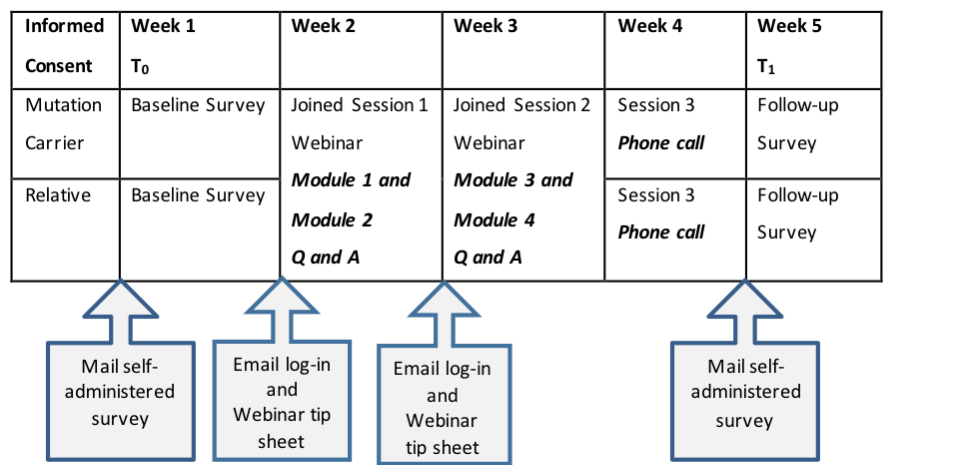
Procedures of the Family Gene Toolkit.

The Family Gene Toolkit is delivered over a period of 4 weeks by two expert clinicians (ie, a certified genetic counselor and a master’s prepared oncology nurse) using two live webinars (PowerPoint presentations with live audio) and one brief follow-up phone call. Dyads log in to a password-protected website synchronously (same time on different computers) to attend the live webinars. The first webinar includes modules 1 and 2, facilitated by a certified genetic counselor. The second is offered a week later; it includes modules 3 and 4, facilitated by a master’s prepared oncology nurse. Each webinar lasts 60 min (45 min presentation and 15 min for questions and answers). A live webinar was considered the optimal mode of delivery because it enabled real-time face-to-face interaction among family members and expert clinicians, enhancing the credibility of the intervention. Family members could easily access the program from home, which is less costly and more convenient than traveling to a clinical site. Convenience and easy access are essential to disseminate the program more widely in the future [33]. Each participant also receives a 15-min phone call with the genetic counselor and the nurse, tailored to address individual concerns (Figure 3).

## Methods

### Study 1: Focus Groups to Assess Acceptability and Participant Satisfaction

After developing the prototype modules, focus groups assessed acceptability and patient satisfaction. Focus groups included women who were older than 18 years and were *BRCA* mutation carriers or female relatives (first- or second-degree, or first cousin) who had not previously received genetic testing. The institutional review board (IRB) of a university-affiliated Comprehensive Cancer Center approved the study. Participants were shown a prototype of the Family Gene Toolkit as a PowerPoint presentation in a 2-hour, face-to-face session. Discussions were audiotaped and transcribed *verbatim*. Team members analyzed transcripts for common responses. A 6-item survey assessed intervention acceptability, ease of use, clarity, appropriate length, level of detail, relevance, interest, and satisfaction (Likert scale 1=low to 7=high) [34,35]. Participants rated their overall satisfaction with the content, the extent it could help with communication and decision making, and the format and appearance of the program.

### Study 2: Pre-Post Pilot to Assess Usability and Feasibility

Suggestions for improvement from the focus groups were incorporated in the prototype intervention. A pre-post pilot study with random allocation to a wait-listed control group was planned to assess usability and feasibility of the updated Family Gene Toolkit delivered in a webinar format (Multimedia Appendix 2). A different certified genetic counselor and master’s prepared oncology nurse were trained to deliver the intervention.

Webinars (PowerPoint presentations with live audio) and phone calls were recorded to assess protocol fidelity. The study was approved by all involved IRBs.

The following sources were used to identify *BRCA* carriers over a period of 18 months: a genetic clinic and the online Clinical Trial Registration Unit from a university-affiliated Comprehensive Cancer Center, a genetic clinic affiliated with a local tertiary hospital, a local online support group and another study assessing use of genetic services in women with early-onset breast cancer [36]. Similar eligibility criteria applied to mutation carriers and relatives: older than 18 years, identified with a pathogenic *BRCA* variant or female relatives (first- or second-degree, or first cousin) who had not undergone genetic testing, carriers willing to invite one female relative, could read and write in English, and provide consent. *BRCA* carriers self-referred to the study were asked to submit a copy of their test results or sign a release form to ascertain their eligibility with the testing company.

*BRCA* carriers received an invitation letter from the medical director of the respective clinic and an informed consent form. When phone numbers were available, invitation letters were followed by a phone call 3 to 4 weeks later. Upon receiving the signed consent, a genetic counselor identified eligible relatives from the carrier’s family history. Carriers received a letter explaining they could invite a relative of their choice among those included in the list. Once both members of the dyad (ie, *BRCA* carrier and relative) returned a signed consent form, they each received a paper and pencil baseline survey. Upon receipt of the completed survey, the webinars and the 15-min phone calls were scheduled. The dyad received via email a link to the webinar, along with information on how to log in to the website. One week after completing the webinars and the phone call, participants received the follow-up survey. Dyads randomly assigned to the wait-listed control group received the baseline and follow-up surveys 4 weeks apart.

Validated instruments assessed family communication, [37] knowledge of breast cancer risk factors [38] and breast cancer genetics [28], perceived breast cancer risk [39], fear of cancer recurrence [40], decisional conflict [41], coping [42], self-efficacy [43] and intention to undergo genetic testing [44,45]. Access to genetic services was assessed with multiple response questions regarding a provider recommendation, eg, *my doctor said I don’t need it*; availability of services, eg, *clinics are too far away*; accessibility of services, *lack of transportation*; and acceptability of services, eg, *I would rather not know if I have a mutation connected to cancer.*

## Results

### Results From Study 1 (Focus Groups)

A purposeful sample of 25 *BRCA* carriers from a genetic risk clinic was invited in the focus groups. Three focus groups were conducted (N=11; 10 mutation carriers and one niece; 44% acceptance rate) to determine the acceptability of the Family Gene Toolkit and participant satisfaction. All 11 participants were white and in the age range of 32 to 60 years (mean age 46, SD 12); most were married or partnered (n=8), college educated (n=9), with an annual family income greater than US $80,000 (n=6). All 11 participants rated their level of comfort and skills using computers as very high (1=low to 7=high; 6.7 [SD 0.48] and 6.1 [SD 0.32], respectively) and their level of comfort and skills using the Internet as very high (1=low to 7=high; 6.6 [SD 0.52], 6.1 [SD 0.57], respectively).

Participants were highly satisfied with the Family Gene Toolkit (6.80 [SD 0.42]), pleased (6.88 [SD 0.35]), and contented (6.63 [SD 0.52]). The content of each module was rated highly on importance and usefulness and was not confusing or did not make participants feel uncomfortable. Participants also reported high satisfaction with the communication module and the decision aid for genetic testing (Multimedia Appendix 3). Participants valued the narratives and testimonials used to illustrate relevant content. They also reported that the intervention could reduce a current gap in health care delivery; it was useful and relevant. Satisfaction with the appearance and length of the modules was high. Participants suggested including more information about testing children, how to support relatives who test negative and husbands, and management of cancer risk. They preferred live webinars involving contact with an expert to a website as a more effective educational tool. However, they thought that scheduling could interfere with the success of this approach. When asked about the best time frame to intervene (eg, immediately after the diagnosis), some participants indicated they would prefer the program immediately after they were identified as *BRCA* carriers, and others thought this would be an added burden. There was no consensus on timing (Multimedia Appendix 4). Information obtained from the focus groups and the content experts was incorporated in the prototype of the intervention.

### Results From Study 2 (Pre-Post Test Pilot)

**Figure 4:**
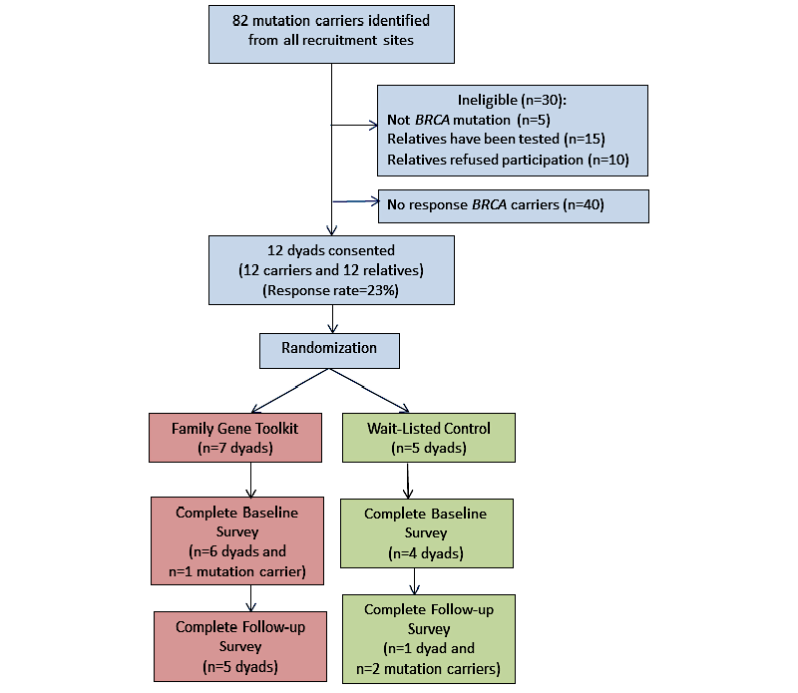
Consolidated Standards of Reporting Trials (CONSORT) diagram for mutation carrier and relative recruitment and random assignment to Family Gene Toolkit versus wait-listed control group. *BRCA*: breast cancer genes.

Over 18 months, 82 potentially eligible mutation carriers were identified for the pre-post pilot study. Some mutation carriers were ineligible to participate (n=30) because they carried another mutation, or because all relatives had been tested or had refused participation. Signed consent forms were returned from 12 mutation carriers (response rate (12/52, 23%) and 12 relatives (12 dyads; n=24). Only first-degree relatives accepted participation (eight sisters; one daughter; one mother). Reasons for relative nonparticipation are unknown as the research team only had direct contact with relatives after they had signed a consent form. Dyads were randomized either to the Family Gene Toolkit (n=7 dyads) or to the wait-listed control (n=5 dyads, see Figure 4).

A completed baseline survey was returned from 10 dyads (n=20) at baseline. All participants were white, in the age range of 8 to 62 years (mean 41, SD 13); most were college educated (n=16), worked full time (n=14), married or partnered (n=11), and with family annual income greater than US $80,000 (n=10). Of the 10 *BRCA* carriers (mean years since genetic testing 4.4, SD 3.2), 4 were diagnosed with invasive breast cancer, 3 with ductal carcinoma *in situ*, 1 with ovarian cancer, and 2 with other forms of cancer.

Carriers were older than relatives (49 [SD 7] vs 34 [SD 3], *t*_2_=2.871, *P*=.01). A completed follow-up survey was returned from 5 dyads in the intervention group and from 1 dyad and two mutation carriers in the wait-listed control group. Completion rates were 71% (10/14) and 20% (2/10) for the intervention and the control groups, respectively (Figure 4). Known reasons for withdrawal were scheduling conflicts (n=3 relatives) and pursuing genetic testing during the intervention (n=1 relative).

We assessed family communication, knowledge of breast cancer risk factors, and breast cancer genetics; coping, perceived breast cancer risk, fear of cancer recurrence and decisional regret in mutation carriers, and decisional conflict, self-efficacy, and intention for genetic testing in relatives (Multimedia Appendix 5). Due to the small sample size, statistical evaluation of intervention effects was not undertaken. However, we evaluated facilitators of genetic testing listed by mutation carriers and relatives. Common facilitators were acceptability of genetic services (eg, *I wanted to know more about my future cancer risk*; n=8), followed by accessibility of services (eg, *my medical insurance covered the cost of the test*; n=4), and availability of services (eg, *the clinic was close to home*; n=2). Barriers for genetic testing for relatives were related to accessibility of genetic services (eg, *I can’t get time off work*; n=4), followed by acceptability of testing (eg, *I would rather not know if I have a mutation connected to cancer*; n=3), and availability of services (eg, *genetic clinics are too far away*; n=1).

## Discussion

### Principal Findings

This paper presents the development and pilot testing of a psycho-educational and skills-building intervention targeting *BRCA* families. The Family Gene Toolkit is designed to provide comprehensive support to *BRCA* families and addresses the challenges faced by mutation carriers and untested relatives. It is a theory-based intervention leveraging the core factual knowledge of biology and medicine and the nondirectionality of genetic counseling. The program also leverages nursing expertise helping patients with a life-threatening diagnosis and addresses needs for family cohesion during times of adversity. Acceptance of the intervention and high participant satisfaction suggests that the Family Gene Toolkit appears to have the potential to meet the needs of these families. However, assessment of acceptability, usability, and feasibility indicated that the method of intervention delivery needed some fine-tuning. The information obtained from the pre-post usability and feasibility studies assisted with further intervention development and testing.

#### Acceptability of the Intervention: Participant Satisfaction Was High

Focus groups valued the Family Gene Toolkit. Participants were highly satisfied with the intervention and reported it was a much-needed service. They were highly satisfied with modules addressing coping and family communication, usefulness, and the completeness of information. Satisfaction was also high with module appearance, formatting, and the quotes used to illustrate pertinent content. These levels of satisfaction suggest that *BRCA* families valued support for decision making, coping, and family communication, in addition to the support they receive from current health care services.

#### Enhancing Usability: The Intervention Is Needed When the Breast Cancer Mutation Is Identified.

Information from about 35% of mutation carriers indicated that “timing” of intervention influenced the usability of the Family Gene Toolkit. Many mutation carriers were not eligible to participate because all their relatives had already been tested. Of the relatives who participated in the pre-post pilot study, none had undergone genetic testing even though the mutation was diagnosed on average 4.4 years previously in their family. Relatives reported that genetic testing was not their priority and that they would rather not know if they had a cancer-predisposing mutation. Relatives who did not accept participation in the study could have possibly refused genetic counseling several times in the past and perhaps were not open to an intervention for family communication, coping, and decision support. These observations suggest that the optimal time for delivering the Family Gene Toolkit is shortly after a positive test result. Future sessions should probably be planned between 3 to 6 months after the *BRCA* mutation is identified. Moreover, prospective recruitment of newly diagnosed *BRCA* families will help identify more mutation carriers whose relatives were not tested and may increase acceptance among relatives who are more open to receiving expert information.

#### Enhancing Feasibility: The Intervention Should Be Delivered as an Asynchronous Website

PtDAs employ various methods for development and evaluation, making comparisons very difficult [46,47]. However, very few PtDAs were developed as interactive Web-based platforms. The growing demand for genetic services makes tele-genetics an attractive option for increasing access, equity, and cost-effectiveness [48]. Technology-enabled genetic counseling is an acceptable option among patients [49], while costs are half those of traditional face-to-face consultations [50]. Web-based PtDAs match face-to-face consultations in both educating patients about genetic screening and decreasing decisional conflict [51,52].

Focus groups indicated that live webinars with certified specialists were credible and reliable sources of information and could provide tailored answers to family members. However, the live webinars have to accommodate participants’ schedules, a significant challenge because of differences in lifestyles and time zones, which in turn affected the feasibility of the intervention. Reconfiguring the Family Gene Toolkit as an “asynchronous” website (ie, participants log in on their own without a live presentation) will also address the issue of optimal timing for intervention delivery by allowing mutation carriers and relatives to access the intervention when they feel ready to discuss the mutation with their family. This will give the families time to consider the decision-making process independent of a specific appointment.

Reconfiguration of the delivery mode has to capture the high relevance of a “live” information-providing session along with ease of using the Web. Two possible approaches for an asynchronous website are envisioned. A targeted version involves recordings of the two webinars and provides all participants with the same information. This approach can be efficacious in increasing knowledge about cancer genetics [53]. A tailor-made approach involves an interactive website that provides information relative to cancer diagnosis, relationship of relative to the mutation carrier, etc. This approach, although more costly to develop initially, was more efficacious with another family- and Web-based intervention [54].

#### Enhancing Recruitment: Personal Contact to Mutation Carriers and Relatives

Although we have successfully used the same recruitment method (patient recruiting relative) in our prior studies targeting women completing genetic testing and young breast cancer survivors [11,14,55], the usability and feasibility study indicated that recruitment of mutation carriers and relatives for a family-based intervention requires personal contact and follow-up phone calls. The pre-post pilot study indicated that personal contact with mutation carriers is a necessary first step to assess their eligibility to participate in the Family Gene Toolkit (ie, confirmed *BRCA* mutation, with not all relatives having been tested). Second, the intervention can help them prepare how to suggest family participation in an intervention study with their relative and help minimize relative refusal rate. Enhanced collaboration with clinicians and clinical settings is expected to help increase participation in a family-based intervention.

### Limitations

The prototype of the Family Gene Toolkit was tested with a homogeneous sample of white, middle to upper class women, recruited from a midwestern US state. Its acceptability and patient satisfaction cannot be guaranteed with diverse and minority families and families from lower socioeconomic status. Recruitment rate among carriers and relatives was lower than expected possibly because of delayed contact (ie, average time postgenetic testing for mutation carriers was 4.4 years, and most of the biological relatives had already undergone genetic testing). Moreover, relatives were significantly younger than mutation carriers, and they may have had specific needs that were not addressed during the recruitment process. Young women at risk of hereditary cancer often have heightened perceptions of risk, chronic depression, and anxiety [56-58], which may interfere with their willingness to participate in the study. Finally, in the prototype model of the Family Gene Toolkit, we focused on *BRCA* pathogenic variants, although panel testing has identified multiple genes associated with hereditary breast and ovarian cancer. *BRCA* mutations are most the commonly associated with HBOC. We developed the prototype addressing the most common pathogenic variants to examine whether this was helpful to mutation carriers and relatives. Modifications include addressing other pathogenic variants and tailoring the Family Gene Toolkit to individuals with other types of cancer and to specific needs of younger women.

### Conclusions

Expanding genetic care has created a need for easy access to this information. Advances in technology are followed by an increase in Web-based health interventions, under the assumption that they provide easy and convenient access to this specialized information [33,59]. Communicating hereditary cancer risks at the familial and professional level poses several challenges both at the medical and social level and requires interprofessional collaboration. The Family Gene Toolkit, though it is not the only PtDA targeting *BRCA* families, addresses the needs of the family as the unit of care. It leverages expertise of a multidisciplinary health care team, which is increasingly recognized as a necessary requirement to address the complex needs of *BRCA* families at the individual, societal, and health policy level. The Family Gene Toolkit is a sustainable Web-based PtDA that can help optimize health care delivery and can greatly contribute to personalized health care.

## Appendix

Multimedia Appendix 1 Patient decision aids for *BRCA* mutation carriers and biological relatives.

Multimedia Appendix 2 Pre-post pilot study design.

Multimedia Appendix 3 Satisfaction with the Family GeneToolkit- Focus groups short survey.

Multimedia Appendix 4 Acceptability of the Family Gene Toolkit - Focus groups interview questions.

Multimedia Appendix 5 Instrument scores in the pre-post pilot study.

## Abbreviations

BRCA: breast cancer genes
HBOC: hereditary breast and ovarian cancer
HER2: human epidermal growth factor receptor 2
IRB: institutional review board
PtDA: patient decision aid
