# Supplementary material for: Development of a Web-based Family Intervention for BRCA Carriers and Their Biological Relatives: Acceptability, Feasibility, and Usability Study
Source: JMIR Cancer. 2018 Apr 13;4(1):e7. doi: 10.2196/cancer.9210 (PMC5924376; doi:10.2196/cancer.9210)
Supplement: Multimedia Appendix 1 [file cancer_v4i1e7_app1.pdf]

Table 1. Patient decision aids (PtDAs) (n=32) for *BRCA* mutation carriers and biological relatives.

First author, year [ref]	Intervention	Mode of delivery	Sample	Findings
Armstrong et al., 2005 [22]	Tailored Decision Support System (DSS) + education booklet vs. education booklet	Face-to-face	32 women, confirmed *BRCA* carriers	• Higher decision satisfaction with DSS at 6 weeks.<br>• No effect on cancer anxiety, perceived cancer risk, management decisions
Bennett et al., 2007 [23]	Distraction-based coping leaflet vs. standard care	Mail	162 women referred for HBOC genetic risk assessment	• No significant reduction in distress at 4-6 week, only among women with high baseline stress
Bloom et al., 2006 [24]	Tailored telephone counseling vs. delayed telephone counseling	Telephone	163 women with a sister diagnosed with breast cancer younger than 50y.o.	• Reduction in overestimation of cancer risk among women older than 50, and increase in physical activity at 6 months.<br>• No change in cancer worries and screening behaviors.
Bodurtha et al. 2014 [25]	KinFact (20 min interactive presentation for individualized breast and colon cancer risk and family communication) vs. handout on lowering breast and colon cancer risk and screening	Face to face	435 women with breast or colon cancer, primary care settings	• KinFact increased gathering of family health history and sharing risk information at 1, 6, and 14 months<br>• KinFact increased frequency of communication about cancer risk at 1, 6, and 14 months<br>• KinFact had better effects among non-pregnant women and women with higher genetic literacy
Bowen et al., 2002 [26]	Individual genetic counseling vs. psychosocial group counseling vs. control	Face-to-face	357 women with at least one relative with breast cancer, but no indication of a germline mutation	• No effect on beliefs about genetic risk or genetic testing

Bowen et al., 2006 [27]	Individual genetic counseling vs. psychosocial group counseling vs. control	Face-to-face	211 Ashkenazi Jewish women with no history of breast or ovarian cancer and no indication of a germline mutation	• Both counseling methods reduced cancer worry, lowered perceptions of breast cancer risk, and decreased interest in genetic testing at 6 months.<br>• No difference between individual and psychosocial counseling group.
Culver et al., 2011 [28]	Web-based PtDA for reducing cancer risk	Focus groups	58 stakeholders, including confirmed *BRCA* female breast cancer survivors, breast cancer advocates, genetics and oncology professionals	• Favorable feedback for PtDA utility, information content, visual display, and output page for values and preferences ranking exercise displaying personalized responses.<br>• No consensus whether the PtDA should be offered at-home or in a clinical setting.
Esplen et al., 2004 [29]	Supportive-expressive writing therapy for 6 months	Face-to-face	70 women, confirmed *BRCA* carriers	• Reduction of cancer worries, anxiety, and depression at 6 months.<br>• No changes in knowledge and surveillance<br>• Increased decisions for prophylactic surgery.
Forrest et al., 2008 [30]	Enhanced counseling for family communication of genetic information vs. control	Face-to-face in a clinic setting	95 biological relatives of *BRCA* carriers and 36 biological relatives at-risk for other genetic conditions (HNPCC, MEN1, Peutz-Jegher syndrome, X-linked condition, balanced reciprocal translocation)	• 61% of biological relatives receiving enhanced counseling contacted a genetic service, vs. 36% in the control group.

Green et al., 2004 [31]	Educational CD-ROM followed by genetic counseling vs. genetic counseling	Computer and/or face-to-face in a clinic setting	211 women with personal or family history of breast cancer	• CD-ROM increased knowledge of breast cancer and genetic testing in women at low-risk for carrying a *BRCA* mutation.<br>• Genetic counseling reduced anxiety and facilitated more accurate risk perceptions.
Hooker et al., 2011 [32]	CD-ROM-based interactive PtDA (CD-ROM PtDA) plus genetic counseling vs. genetic counseling	CD-ROM, home setting	214 women, confirmed *BRCA* carriers	• CD-ROM-PtDA decreased cancer-specific distress and genetic testing-specific distress.<br>• The overall decrease in distress between the two groups was similar at 12 months.
Joseph et al., 2010 [33]	Cancer Risk Education Intervention Tool (CREdIT), computer-based, non-interactive slide presentation	Face-to face in a clinic setting	Women referred for HBOC risk assessment; 52 for pilot testing and 11 for interviews	• CREdIT presented new concepts; had appealing format; no effect on perceived risk; no effect on knowledge; increased preparedness for genetic counseling.
Juan et al., 2008 [34]	Patient decision aid (PtDA) booklet for men	Mail	27 men who received genetic testing for *BRCA* mutations	• PtDA booklet was acceptable and patient satisfaction was high.<br>• PtDA booklet increased knowledge and value-based preferences about genetic testing.
Kaphingst et al., 2009 [35]	Virtual reality active learning vs. traditional didactic learning for genetic concepts	Face-to-face	156 adults without specialized genetic knowledge	• Traditional didactic learning increased recall and believability, and had greater impact on mental models<br>• Virtual reality active learning increased motivation, interest, and enjoyment.
Kardashian et al., 2012 [36]	Sharing Risk Information Tool (ShaRIT), personalized	Face-to-face in a	19 women, confirmed *BRCA* carriers	• ShaRIT was a useful resource.

	education (binder, CD) + genetic counseling vs. genetic counseling	clinic setting		• ShaRIT increased family communication of genetic test results.<br>• Female gender, degree of relationship, and frequency of communication influenced family communication of test results.
Lerman et al. 1997 [37]	Education vs. education + genetic counseling vs. control group	Face-to-face in clinic setting	578 women referred for *BRCA* testing	• Education and education + genetic counseling increased genetic knowledge<br>• Education + counseling increased perceived limitations and risks of *BRCA* testing and decreased perceived benefits.<br>• Neither approach changed intentions for *BRCA* testing.
Mancini et al. 2006 [38]	Patient Information Booklet (PIB) for *BRCA* testing vs. usual care	Face-to-face in clinic setting	560 female breast cancer patients evaluated for HBOC risk	• PIB increased satisfaction with information, decreased decisional conflict due to lack of information, and increased rates of genetic testing.<br>• PIB marginally increased knowledge.
Matloff et al. 2006 [39]	Personalized risk assessment and genetic counseling for treatment of menopausal symptoms vs. usual care	Face-to-face in clinic setting	48 cancer-free, female, first degree relatives of breast cancer patients	• Intervention increased knowledge at 1 and 6 months.<br>• Perceived breast cancer risk and perceived heart disease risk were more accurate at 1 and 6 months.<br>• Reduced intention for menopausal treatment in both groups.
McInerney-Leo et al. 2004 [40]	Problem-solving training (PST) vs. client-centered psychological counseling	Face-to-face in a clinic setting	212 untested female biological relatives of *BRCA* carriers	• PST increased psychological well-being and reduced depressive symptoms among relatives who

				chose to be tested at 6 and 9 months.
McKinnon et al. 2007 [41]	One-day retreat with education about medical management, genetic privacy and discrimination, and psychological and family issues.	Face-to-face in a community setting	41 confirmed *BRCA* carriers<br>At least 30 biological relatives<br>28 participants completed pre- and post-survey	• Positive evaluations of the retreat.<br>• Improved lifestyle, cancer screening, increased rates of chemoprevention uptake, and planned to have preventive surgery at 6 months.
Miller et al. 2005 [42]	Enhanced counseling (EC) for risk reduction options for ovarian cancer vs. general health information.	Face-to-face in a clinic setting	77 women undergoing *BRCA* testing	• EC reduced avoidance 1 week after receiving genetic test results.<br>• EC increased information seeking for prophylactic oophorectomy and preventive surgery at 6-months.
Montgomery at al. 2013 [43]	Communication Skills-Building Intervention (CSBI) vs. nutrition and exercise information.	Face-to-face in a clinic setting	249 women, confirmed *BRCA* carriers	• CSBI did not affect percent of mutation carriers sharing test results with first degree relatives or level of distress associated with genetic testing.
Roussi et al. 2010 [44]	Enhanced counseling (EC) promoting cognitive and affective processing vs. counseling + a general health information session.	Face-to-face in a clinic setting	134 women prior to genetic testing	• EC increased knowledge at 1 week after disclosure of test results.<br>• EC reduced distress for women who tested positive at 1 week after disclosure of test results.

Rupert et al. 2013 [45]	Cancer in the Family, (CitF) - online clinical decision support tool	Face-to-face in primary care clinics	9 primary healthcare providers and 48 women with no personal history of cancer	• CitF collected complete family histories (67%), calculated personal breast cancer risk (96%), and facilitated sharing printouts with providers (65%).<br>• CitF increased HBOC knowledge and prompted patent-provider discussions about HBOC risk and cancer family history.
Santerre-Theil et al. 2016 [46]	Communication guidance booklet (CGB) for sharing test results with underage children	Focus groups and in-depth interviews	9 female confirmed *BRCA* carriers and 3 genetic specialists	• CGB was acceptable.
Schackmann et al. 2013 [47]	Online PtDA for cancer risk reduction	Online for PtDA testing and face-to-face for evaluation	40 female, confirmed *BRCA* carriers and 16 clinicians	• PtDA was easy to use, and general satisfaction was high.<br>• PtDA was usable and clinically relevant.
Skinner et al. 2002 [48]	Tailored Print Materials (TPMs) about genetic testing vs. Non-Tailored Print Material (NTPMs)	Mail	262 women with family history of breast or ovarian cancer and greater than 10% probability of carrying a *BRCA* mutation	• TPMs increased knowledge and improved accuracy of perceived risk of being a mutation carrier<br>• TPMs did not reduce anxiety
Tiller et al. 2006 [49]	Tailored Decision Aid (TDA) (booklet and exercise for values clarification) for managing ovarian cancer risk vs. general educational pamphlet	Mail	131 women with family history of breast and/or ovarian cancer or hereditary nonpolyposis colorectal cancer	• TDA had high acceptability rate and did not increase psychological distress.<br>• TDA decreased decisional conflict and increased knowledge about risk management options at 2 weeks but not at 6 months.

Van Roosmalen et al. 2004 [50]	Shared decision-making intervention (SDMI) for screening vs. prophylactic surgery for breasts and/or ovaries.	Face-to-face in a clinic setting	88 women, confirmed *BRCA* carriers	• SDMI had no effect on treatment choice, but enhanced values clarification at 3 and at 9 months.<br>• SDMI reduced intrusive thoughts and depression at 9 months.<br>• SDMI interacted with cancer history, benefiting unaffected women at 9 months.
Venne and Hamann 2007 [51]	Peer education with genetic component vs. peer education without genetic component	Face-to-face	88 women with breast cancer	• Genetic module increased knowledge.<br>• Interest in genetic testing was not different between the two groups.
Wakefield et al. 2008 [52]	PtDA for genetic testing (printed material) vs. control pamphlet	Face-to-face in a clinic setting	145 women after genetic counseling for HBOC risk	• PtDA increased knowledge and helped with value clarification.<br>• PtDA had no effect on informed choice, post-decisional regret or decision for genetic testing.<br>• PtDA women were less likely to share information with family members.
Wang et al. 2005 [53]	Educational CD-ROM vs.<br>feedback checklist to genetic counselor vs. CD-ROM + feedback checklist to genetic counselor vs. standard care	CD-ROM and face-to-face in a clinic setting	197 women before genetic assessment for HBOC risk	• CD-ROM reduced face-to-face time with genetic counselor and rates of genetic testing uptake.<br>• Feedback checklist to the genetic counselor increased knowledge of genetics and breast cancer.

References

22. Armstrong K, Weber B, Ubel PA, Peters N, Holmes J, Schwartz JS: Individualized survival curves improve satisfaction with cancer risk management decisions in women with BRCA1/2 mutations. J Clin Oncol 2005, 23(36):9319-9328.

23. Bennett P, Phelps C, Brain K, Hood K, Gray J: A randomized controlled trial of a brief self-help coping intervention designed to reduce distress when awaiting genetic risk information. J Psychosom Res 2007, 63(1):59-64.

24. Bloom JR, Stewart SL, Chang S, You M: Effects of a telephone counseling intervention on sisters of young women with breast cancer. Prev Med 2006, 43(5):379-384.

25. Bodurtha JN, McClish D, Gyure M, Corona R, Krist AH, Rodríguez VM, Maibauer AM, Borzelleca Jr J, Bowen DJ, Quillin JM: The KinFact intervention–A randomized controlled trial to increase family communication about cancer history. Journal of Women's Health 2014, 23(10):806-816.

26. Bowen DJ, Burke W, Yasui Y, McTiernan A, McLeran D: Effects of risk counseling on interest in breast cancer genetic testing for lower risk women. Genet Med 2002, 4(5):359-365.

27. Bowen DJ, Burke W, Culver JO, Press N, Crystal S: Effects of counseling Ashkenazi Jewish women about breast cancer risk. Cultur Divers Ethnic Minor Psychol 2006, 12(1):45-56.

28. Culver JO, MacDonald DJ, Thornton AA, Sand SR, Grant M, Bowen DJ, Burke H, Garcia N, Metcalfe KA, Weitzel JN: Development and evaluation of a decision aid for BRCA carriers with breast cancer. J Genet Couns 2011, 20(3):294-307.

29. Esplen MJ, Hunter J, Leszcz M, Warner E, Narod S, Metcalfe K, Glendon G, Butler K, Liede A, Young MA: A multicenter study of supportive-expressive group therapy for women with BRCA1/BRCA2 mutations. Cancer 2004, 101(10):2327-2340.

30. Forrest LE, Burke J, Bacic S, Amor DJ: Increased genetic counseling support improves communication of genetic information in families. Genet Med 2008, 10(3):167-172.

31. Green MJ, Peterson SK, Baker MW, Harper GR, Friedman LC, Rubinstein WS, Mauger DT: Effect of a computer-based decision aid on knowledge, perceptions, and intentions about genetic testing for breast cancer susceptibility: a randomized controlled trial. JAMA 2004, 292(4):442-452.

32. Hooker GW, Leventhal KG, DeMarco T, Peshkin BN, Finch C, Wahl E, Joines JR, Brown K, Valdimarsdottir H, Schwartz MD: Longitudinal changes in patient distress following interactive decision aid use among BRCA1/2 carriers: a randomized trial. Med Decis Making 2011, 31(3):412-421.

33. Joseph G, Beattie MS, Lee R, Braithwaite D, Wilcox C, Metrikin M, Lamvik K, Luce J: Pre-counseling education for low literacy women at risk of Hereditary Breast and Ovarian Cancer (HBOC): patient

experiences using the Cancer Risk Education Intervention Tool (CREdIT). J Genet Couns 2010, 19(5):447-462.
34. Juan AS, Wakefield CE, Kasparian NA, Kirk J, Tyler J, Tucker K: Development and pilot testing of a decision aid for men considering genetic testing for breast and/or ovarian cancer-related mutations (BRCA1/2). Genet Test 2008, 12(4):523-532.
35. Kaphingst KA, Persky S, McCall C, Lachance C, Loewenstein J, Beall AC, Blascovich J: Testing the effects of educational strategies on comprehension of a genomic concept using virtual reality technology. Patient Educ Couns 2009, 77(2):224-230.
36. Kardashian A, Fehniger J, Creasman J, Cheung E, Beattie MS: A Pilot study of the Sharing Risk Information Tool (ShaRIT) for Families with Hereditary Breast and Ovarian Cancer Syndrome. Hered Cancer Clin Pract 2012, 10(1):4.
37. Lerman C, Kerner J, Gomez-Caminero A, Hughes C, Reed MM, Biesecker B, Benkendorf JL: Controlled trial of pretest education approaches to enhance informed decision-making for BRCA1 gene testing. J Natl Cancer Ins 1997, 89(2):148-157.
38. Mancini J, Nogues C, Adenis C, Berthet P, Bonadona V, Chompret A, Coupier I, Eisinger F, Fricker JP, Gauthier-Villars M et al: Impact of an information booklet on satisfaction and decision-making about BRCA genetic testing. Eur J Cancer 2006, 42(7):871-881.
39. Matloff ET, Moyer A, Shannon KM, Niendorf KB, Col NF: Healthy women with a family history of breast cancer: impact of a tailored genetic counseling intervention on risk perception, knowledge, and menopausal therapy decision making. J Womens Health 2006, 15(7):843-856.
40. McInerney Leo A, Biesecker BB, Hadley DW, Kase RG, Giambarresi TR, Johnson E, Lerman C, Struewing JP: BRCA1/2 testing in hereditary breast and ovarian cancer families: effectiveness of problem-solving training as a counseling intervention. Am J Med Genet A 2004, 130a(3):221-227.
41. McKinnon W, Naud S, Ashikaga T, Colletti R, Wood M: Results of an intervention for individuals and families with BRCA mutations: a model for providing medical updates and psychosocial support following genetic testing. J Genet Couns 2007, 16(4):433-456.
42. Miller SM, Roussi P, Daly MB, Buzaglo JS, Sherman K, Godwin AK, Balshem A, Atchison ME: Enhanced counseling for women undergoing BRCA1/2 testing: impact on subsequent decision making about risk reduction behaviors. Health Educ Behav 2005, 32(5):654-667.
43. Montgomery SV, Barsevick AM, Egleston BL, Bingler R, Ruth K, Miller SM, Malick J, Cescon TP, Daly MB: Preparing individuals to communicate genetic test results to their relatives: report of a randomized control trial. Fam Cancer 2013, 12(3):537-546.

44. Roussi P, Sherman KA, Miller S, Buzaglo J, Daly M, Taylor A, Ross E, Godwin A: Enhanced counselling for women undergoing BRCA1/2 testing: impact on knowledge and psychological distress–results from a randomised clinical trial. Psychol Health 2010, 25(4):401-415.

45. Rupert DJ, Squiers LB, Renaud JM, Whitehead NS, Osborn RJ, Furberg RD, Squire CM, Tzeng JP: Communicating risk of hereditary breast and ovarian cancer with an interactive decision support tool. Patient Educ Couns 2013, 92(2):188-196.

46. Santerre-Theil A, Bouchard K, St-Pierre D, Drolet AM, Chiquette J, Dorval M: Development of a Tool to Guide Parents Carrying a BRCA1/2 Mutation Share Genetic Results with Underage Children. J Cancer Educ 2016.

47. Schackmann EA, Munoz DF, Mills MA, Plevritis SK, Kurian AW: Feasibility evaluation of an online tool to guide decisions for BRCA1/2 mutation carriers. Fam Cancer 2013, 12(1):65-73.

48. Skinner CS, Schildkraut JM, Berry D, Calingaert B, Marcom PK, Sugarman J, Winer EP, Iglehart JD, Futreal PA, Rimer BK: Pre-counseling education materials for BRCA testing: does tailoring make a difference? Genet Test 2002, 6(2):93-105.

49. Tiller K, Meiser B, Gaff C, Kirk J, Dudding T, Phillips K-A, Friedlander M, Tucker K: A randomized controlled trial of a decision aid for women at increased risk of ovarian cancer. Med Decis Making 2006, 26(4):360-372.

50. Van Roosmalen M, Stalmeier P, Verhoef L, Hoekstra-Weebers J, Oosterwijk J, Hoogerbrugge N, Moog U, van Daal W: Randomized trial of a shared decision-making intervention consisting of trade-offs and individualized treatment information for BRCA1/2 mutation carriers. J Clin Oncol 2004, 22(16):3293-3301.

51. Venne VL, Hamann HA: Successful use of peer educators for sharing genetic information. J Genet Couns 2007, 16(4):515-525.

52. Wakefield CE, Meiser B, Homewood J, Peate M, Taylor A, Lobb E, Kirk J, Young MA, Williams R, Dudding T et al: A randomized controlled trial of a decision aid for women considering genetic testing for breast and ovarian cancer risk. Breast Cancer Res Treat 2008, 107(2):289-301.

53. Wang C, Gonzalez R, Milliron KJ, Strecher VJ, Merajver SD: Genetic counseling for BRCA1/2: a randomized controlled trial of two strategies to facilitate the education and counseling process. Am J Med Genet A 2005, 134A(1):66-73.
