# Supplementary material for: Development of a Web-based Family Intervention for BRCA Carriers and Their Biological Relatives: Acceptability, Feasibility, and Usability Study
Source: JMIR Cancer. 2018 Apr 13;4(1):e7. doi: 10.2196/cancer.9210 (PMC5924376; doi:10.2196/cancer.9210)
Supplement: Multimedia Appendix 2 [file cancer_v4i1e7_app2.pdf]

Figure 4: Pre-Post Pilot Study Design

<table>
<tr><th>Informed Consent</th><th>Week 1 T_0</th><th>Week 2</th><th>Week 3</th><th>Week 4</th><th>Week 5 T_1</th><th>Week 6</th><th>Week 7</th><th>Week 8</th></tr>
<tr><td>Family Gene Toolkit Mutation Carrier</td><td>Baseline Survey</td><td rowspan="2">Session 1 Webinar</td><td rowspan="2">Session 2 Webinar</td><td>Session 3 Phone call</td><td>Follow-up Survey</td><td rowspan="2"></td><td rowspan="2"></td><td rowspan="2"></td></tr>
<tr><td>Family Gene Toolkit Relative</td><td>Baseline Survey</td><td>Session 3 Phone call</td><td>Follow-up Survey</td></tr>
<tr><td>Wait-listed Control Mutation Carrier</td><td>Baseline Survey</td><td rowspan="2"></td><td rowspan="2"></td><td rowspan="2"></td><td>Follow-up Survey</td><td rowspan="2">Session 1 Webinar</td><td rowspan="2">Session 2 Webinar</td><td>Session 3 Phone call</td></tr>
<tr><td>Wait-listed Control Relative</td><td>Baseline Survey</td><td>Follow-up Survey</td><td>Session 3 Phone call</td></tr>
</table>
