# Supplementary material for: Development of a Web-based Family Intervention for BRCA Carriers and Their Biological Relatives: Acceptability, Feasibility, and Usability Study
Source: JMIR Cancer. 2018 Apr 13;4(1):e7. doi: 10.2196/cancer.9210 (PMC5924376; doi:10.2196/cancer.9210)
Supplement: Multimedia Appendix 3 [file cancer_v4i1e7_app3.pdf]

Table 2. Satisfaction with the Family GeneToolkit-focus groups short survey (n=11 participants: 10 mutation carriers; 1 relative).

Category	Survey—satisfaction with Family Gene Toolkit					
	Item		Response			
			Frequency (n)			Mean (SD) 1 (low) to 7 (high)
			Not satisfied	Neutral	Satisfied	
**Overall satisfaction**	**How do you feel about your overall experience with this program**					
		Dissatisfied-Satisfied	0	0	11	6.80 (0.42)
		Displeased-Pleased	0	2	9	6.88 (0.35)
		Frustrated-Contented	0	2	9	6.63 (0.52)
**Content**	**Important**					
		The information I received in the program was important to me	0	0	11	7.0 (0.0)
	**Useful**					
		The program was a useful way to learn about genetic mutations	0	0	11	6.6 (0.52)
		The program was a useful way to learn about my cancer risk	0	0	11	6.6 (0.70)
		The program helped me learn ways to manage my cancer risk	1	0	10	5.8 (1.23)
	**Confusing**					
		The information I received in the program was confusing	0	0	11	1.0 (0.0)
		I thought that the program was easy to understand	0	0	11	6.9 (0.32)
		The program was too complex	0	0	11	1.1 (0.32)
	**Uncomfortable**					

		The information in the program made me feel uncomfortable	0	0	11	1.2 (0.42)
**Helpful for family communication**	The program made me think about ways to help my family		0	0	11	6.4 (0.84)
	The program helped me learn how to communicate with my family about cancer		0	0	11	6.4 (0.70)
**Helpful for decision making for genetic testing**	The program made me feel more satisfied with my decision or The program helped me make up my mind about genetic testing		0	1	10	6.5 (1.08
