# Supplementary material for: Development of a Web-based Family Intervention for BRCA Carriers and Their Biological Relatives: Acceptability, Feasibility, and Usability Study
Source: JMIR Cancer. 2018 Apr 13;4(1):e7. doi: 10.2196/cancer.9210 (PMC5924376; doi:10.2196/cancer.9210)
Supplement: Multimedia Appendix 4 [file cancer_v4i1e7_app4.pdf]

Table 3. Acceptability of the Family GeneToolkit-focus groups interview questions (n=11 participants: 10 mutation carrier; 1 relative).

| Theme | Interview questions | Excerpts from focus group participants |
|---|---|---|
| Overall satisfaction | Overall, what do you think about the information covered? | *“...those quotes, those actual experiences, were so eye-opening for me. I was like, wow!”*<br>*“...depends on [the] audience. Researchers want everything. But some people don’t want in detail. Tell me what I have to know.”* |
| | What did you like or dislike the most? | *“That is a little shallow...Maybe that is an issue in some families, though. It wouldn’t be in mine...I have a hard time [believing that] anybody would ever think those things. Who would think someone’s going to blame me? Just, in my situation, I just can’t see these families saying this.”* |
| Content | What did you value the most from the content we covered? | *“...finding this info is very freeing...just knowing that there was a genetic reason why my cancer occurred, was very freeing for me. Certainly quelled the anger factor a bit. And it helps to explain my father’s death, my grandmother’s death, and this long history of early deaths in that side of the family.”* |
| | Do you think the intervention is useful and relevant to the care you received? | *“...the coping module was extremely significant...I’ve been on the cancer world for a very long time. That was the first time I’d seen that information presented that way...it was extremely helpful and one of the best I’ve seen...”* |
| | | *“...this is the information that you want your family members to have...”* |
| | We used the term “damaged gene.” Was it confusing? | *“I liked it because “mutation” sounds different, it sounds painful, there’s something wrong with you.”* |
| | | *““Damaged” makes it less dangerous sounding. Like, you can deal with it.”*<br>*“ It depends on [the] slide. If the information is from [a health care] provider, you sound natural.”* |
| Helpful for family communication | What did you think about the communication module? | *“...what different scenarios can develop when you’re talking with family...it’s important to give the information and not tell them, or expect them to tell you, how they’re going to use it. You can’t control how your family member is going to use the information. That’s their decision.”*<br><br>*“I like how you say that there’s a duty [to share genetic test results]. It’s a responsibility, regardless how it’s fallen on your lap.”*<br><br>*“I really loved the slide that said that they [relatives] also have a responsibility, that it’s not all on you...There is cohesiveness because of it... when the whole family knows, they can support each other, and you address it, but it’s even bigger than that...you’re facing something severe and dangerous. It’s nice to know you have some control and some power...”* |

| | | |
|---|---|---|
| Helpful for decision- making for genetic testing | Regarding the pros and cons of genetic testing, do you think we covered most of them or at least the main ones? | *"...it pretty much tracks my decision-making process. Certainly weighed most of the pros and cons...These were pretty much the factors we weighed in our decision-making process. "*<br><br>*"I don't know if the pros are strong enough. My sister was tested after myself and when she had her ovaries removed, they had cancerous changes. When my niece was tested...positive she decided to have her children younger. So the pros are kind of nice but maybe they could be more assertive."*<br><br>*"I like this [decision making worksheet]. We can do our own...We can discuss how they [relatives] feel, how they rate those options."* |
| Format (appearance, length) | If this program was offered in different formats, which ones would you prefer? | Internet: 6[a]<br>CD or DVD: 5<br>In-person: 2<br>Any format: 1 |
| | What did you think of the presentation? | *"I liked the way they [slides] looked. Didn't lose my attention."* |
| Mode of delivery | What do you think is the best way to deliver this intervention? Do you think that a webinar and logging on at the same time is feasible, considering distance and time zones? Or would it better to record the webinar and the family members can view this information in their own time? | *"Probably that [recorded presentations]...Getting everyone together is always hard...if we could provide them [relatives] with links that they could go to online so that they could review information, and maybe educate themselves more and understand better, then they would go and take the steps to get tested."*<br><br>*"My nieces are busy, they're doing this and that, but if I...say "look, take a few minutes, you can go online, you can look at it, and get additional information, it's all in one place, and you could do it during your own time" they might be more apt to do it."*<br><br>*"I like the idea of a live person. [It is] not effective alone, [it is a] better impression..."* |

| | | |
|---|---|---|
| Missing information | Is there additional information we need to address? | *"[Explaining probabilities and risk] was very important...in my decision-making process. How pathogenic is pathogenic? Am I closer to inconclusive? Or, am I high on the "this is a bad thing to have" scale...Another graphic for women with mutation."*<br><br>*"...one slide talking to my children. When should [we] tell them...when should [we have] them get tested?"*<br><br>*"...it would be really neat in the coping module to speak to the unique challenges faced by spouses, and/or parents. Certainly, my cancer was much more difficult emotionally for my husband and my mom."*<br><br>*"Certainly, for males—our fathers, husbands, and sons—they feel it's a woman's world...Maybe in some pictures you can incorporate some men so that...they have [the] sense that they belong, it's not just women talking to women."*<br><br>*"...about open communication, make the pictures more general, include more men. This is really hard on husbands...on marriages. Any kind of feminine cancer takes a toll on a marriage."*<br><br>*"...there is nothing, if tested negative, what is next?"* |
| Timing of intervention | When is the best time to deliver this information? | *"I guess it depends. For me I wasn't going through cancer treatment. I would say like a week later."*<br><br>*"The way I'm wired, I would've said Yes, sign me up, right away."*<br><br>*"I would've waited...I was going through a lot of different things. I would've been like Oh, I've got too much right now."*<br><br>*"...maybe a month or two, after my major treatment was over."* |
