# Supplementary material for: Development of a Web-based Family Intervention for BRCA Carriers and Their Biological Relatives: Acceptability, Feasibility, and Usability Study
Source: JMIR Cancer. 2018 Apr 13;4(1):e7. doi: 10.2196/cancer.9210 (PMC5924376; doi:10.2196/cancer.9210)
Supplement: Multimedia Appendix 5 [file cancer_v4i1e7_app5.pdf]

Table 4. Instrument scores in pre-post intervention survey.

Measures	Experimental (n=10)				Control (n=4)			
	Mutation carriers (n=5)		Relatives (n=5)		Mutation carriers (n=3)		Relative (n=1)	
	T_0, mean (SD)	T_1, mean (SD)	T_0, mean (SD)	T_1, mean (SD)	T_0, mean (SD)	T_1, mean (SD)	T_0	T_1
Mutuality interpersonal sensitivity	4.64 (0.59)	4.38 (0.36)	4.96 (0.40)	4.84 (0.51)	4.17 (1.29)	4.38 (1.39)	4.50	4.97
Knowledge of risk factors	14.00 (1.41)	13.60 (3.21)	7.80 (4.38)	12.60 (3.51)	15.67 (1.53)	16.00 (1.00)	14.00	10.00
Knowledge of cancer genetics	11.20 (0.84)	11.80 (1.30)	6.60 (3.36)	10.80 (2.17)	9.67 (0.58)	10.33 (0.58)	4.00	2.00
Brief COPE	5.10 (0.58)	5.09 (0.45)	4.93 (0.49)	5.08 (0.59)	4.66 (0.61)	5.20 (0.68)	5.22	5.07
Perceived risk	6.40 (3.21)	5.20 (2.28)	4.80 (1.92)	5.60 (1.95)	6.33 (0.58)	6.67 (1.53)	5	7
Fear of cancer recurrence[a]	3.56 (0.75)	3.88 (0.78)	-	-	4.75 (2.12)	5.25 (0.71)	-	-
Cancer worry	-	-	5.76 (2.31)	5.66 (1.60)	-	-	3.14	3.14
Decisional regret scale	1.20 (0.45)	1.12 (0.18)	-	-	1.00 (0.00)	1.13 (0.12)	-	-
Decisional conflict scale	-	-	4.22 (0.93)	4.31 (0.43)	-	-	4.36	4.21
Self-efficacy genetic testing	-	-	5.40 (1.52	5.20 (2.49)	-	-	5.00	6.00
Intention for genetic testing	-	-	2.60 (2.07)	1.40 (0.55)	-	-	3.00	2.00

[a]Sample size=6 probands (4 experimental and 2 control).
